# The effects of cognitive effort on academic performance of learners with cochlear implants in a private mainstream school in Gauteng

**DOI:** 10.4102/ajod.v11i0.886

**Published:** 2022-10-28

**Authors:** Lior Blumenthal, Maximus M. Sefotho

**Affiliations:** 1 Department of Educational Psychology, Faculty of Education, University of Johannesburg, Johannesburg, South Africa

**Keywords:** Auditory challenges, cognitive effort, cochlear implants, cognitive effort fatigue, disability, phenomenology

## Abstract

**Background:**

This research investigated the phenomenon of learners with cochlear implants and their challenges with cognitive effort in private mainstream schools in Gauteng. Many learners with cochlear implants encounter academic and social challenges at school, despite the advanced technology.

**Objectives:**

This study aimed to explore how learners with cochlear implants experience cognitive effort and whether it impacts their academic potential.

**Methods:**

Research was conducted using a phenomenological design. Phenomenography was used as theoretical framework to perceive, interpret and understand experiences of the cochlear implant recipients. The six former learners who were recipients of cochlear implants were selected using purposive sampling. Semistructured interviews were utilised to gather information, which was analysed using thematic content analysis.

**Results:**

Five themes emerged from the analysis, namely auditory challenges, cognitive functioning, peer interactions, emotional health and concealed disability. This article only presents the first theme of cognitive functioning and highlights three subthemes related to cognitive effort. Findings show that many learners struggled with their concentration span and fatigue, as a result of their cognitive effort difficulties.

**Conclusion:**

This study demonstrated how learners with cochlear implants face challenges with cognitive effort at their mainstream schools. It indicates the need for awareness of and training on educating learners with cochlear implants to help them reach their academic potential.

**Contribution:**

This study contributes a unique focus on learners with cochlear implants in mainstream schools in South Africa. The study highlights that cognitive effort of learners with cochlear implants influenced their capabilities to multitask and retain information, despite the effort they have to put into listening. Further research should be conducted to develop interventions that could lesson cognitive effort while increasing learner productivity. The article responds to disability studies and inclusive education.

## Introduction

This article investigated gaps in the literature on mainstream education for learners with cochlear implants regarding cognitive effort. The objective was to obtain insight into the experiences and challenges of cognitive effort, which learners with cochlear implants experience in South African mainstream schools. Furthermore, this article sought out to explore the various factors that contribute to the challenges of cognitive effort for these learners.

Globally, there is limited research on the cognitive effort of learners with cochlear implants. There is a gap in literature on the challenges that learners with cochlear implants face with cognitive effort in South Africa. This lack of data highlights the significance of this study because it examines the reasons for the cognitive effort challenges that learners with cochlear implants experience.

Cognitive effort refers to the amount of thinking and interpretation required in order to decipher verbal information (Van Trijp 2016). Westbrook and Braver (2015:395), equated cognitive effort with ‘effort-based decision-making’ on the auditory information received in class for effective meaning-making. Cognitive effort assists with optimisation of the information received in order to allow learners with cochlear implants to succeed in their studies (Kuldas et al. 2014). Learners with cochlear implants communicate that they need additional cognitive effort to interpret spoken information, and therefore, this impacts their capacity to do more tasks at one time and hold onto information (Purdy et al. 2017). More effort is required to decode spoken information in the classroom. When their cognitive effort is compromised, it is harder to concentrate in class. This therefore affects their academic capabilities. This study set out to establish the impact of cognitive effort on learners with cochlear implants. Cognitive effort manifests itself in various ways.

This study investigated ways to ascertain that learners have the support and guidance they require to achieve their academic potential. Hearing impairments and their effect on the academic potential of learners in mainstream schools is the main focus of South African literature (Kemp, Skrebneva & Krüger 2011; Skrebneva 2010). This research attempted to bridge the gap regarding understanding of the challenges with cognitive effort that learners with cochlear implants experience.

The study aligns with the inclusion mandate of South Africa through focused projects such as the Centre for Deaf Studies at the University of Witwatersrand. The centre advocates for ‘moving beyond hearing screening’ (Störbeck & Pittman 2008:36) to inclusion of learners with disabilities from early childhood development (ECD) (Storbeck & Moodley 2011). This addresses the national policy on inclusion, the Education White Paper 6 (Du Plessis 2013) and the Sustainable Development Goals: (1) Goal 3, good health and well-being; (2) Goal 4, quality education; and (3) Goal 10, reduced inequalities (Haywood et al. 2019), with a view to specifically include learners with cochlear implants.

The cochlear implant is an advanced and sophisticated technology that is used to provide hearing abilities for individuals who are profoundly hearing impaired. It is an artificial device that improves hearing by utilising electrical stimulations (Piotrowska, Paradowska-Stankiewicz & Skarżyński 2017). It consists of an external device (Briggs 2011) and internal components that operate in the inner ear (Hainarosie, Zainea & Hainarosie 2014). The cochlear implant provides enough sounds and frequencies for the recipient to hear language, speech and environmental sounds for the recipient, but it does not restore the auditory sense (Joseph & Lassen 2013). The aim and purpose of the cochlear implant is to increase the hearing sense.

Cochlear implants have been available for learners who are hearing impaired since the 1990s, and many opportunities that were unavailable to them have now become accessible to them (Fitzpatrick & Olds 2015). As a result of this technology, many learners with cochlear implants are able to experience the realm of the auditory sense and can also learn and establish spoken language (Vermeulen et al. 2012). Cochlear implants have generally enabled learners who are hearing impaired to access mainstream schooling (De Raeve 2014). In South Africa at present, many learners with cochlear implants are enrolled at mainstream schools rather than attending specialised schools for learners who are hearing impaired (Takala & Sume 2018).

However, many learners with cochlear implants face difficulties within their mainstream schools (Diaz et al. 2019; Punch & Hyde 2010). Learners with cochlear implants still encounter challenges of maintaining a similar pace to their hearing peers at school, despite the advancement in their hearing and speech. (Marschark et al. 2019; Punch & Hyde 2010). These challenges include extended demands on their listening skills and working harder than their hearing peers to decode spoken information, especially when their teachers have accents different from their own. A factor that influences learners with cochlear implants in mainstream schools is cognitive effort.

### Theoretical orientation

Phenomenology was used as a theoretical framework that oriented this study (Larsen & Adu 2021). The phenomenological framework guided the study in the interpretation and understanding of lived experiences of the cochlear implant recipients (Yüksel & Yıldırım 2015). Phenomenology as a theoretical framework in this study served to anchor the investigation firmly in literature and linking to the results. Marton (1981:180) described phenomenology as the ‘description, analysis and understanding of experiences, that is, research which is directed towards experiential description’. As a theoretical framework in this study, phenomenology helped to frame the researchers’ understanding of ‘ways in which people experience, interpret, understand, perceive or conceptualise a certain cognitive effort of learners with cochlear implants’ (Orgill 2012:2608). As listening becomes a demanding cognitive task for learners with cochlear implants, cognitive effort can be perceived to alleviate probable cognitive dissonance (Vaidis & Bran 2019). In agreement with Grant and Osanloo (2014), phenomenology could be considered a theoretical framework or blueprint that ontologically, philosophically, epistemologically and methodologically resonates with the interpretive paradigm and phenomenological design of this study.

## Research methods and design

This study utilised a qualitative research method based on the phenomenological design (Kafle 2011; Khan 2014; Ratislavová & Ratislav 2014). According to Busetto, Wick and Gumbinger (2020), qualitative research can be defined as the study of the nature of phenomena. In this study, phenomena studies centred on exploring how learners with cochlear implants experience cognitive effort and whether it impacts their academic potential. This provided the opportunity for the research process to be investigative and analytical (Campbell 2014). In addition, the study utilised the phenomenological research design (Kafle 2011) to investigate the cognitive effort of learners with cochlear implants at mainstream schools. The phenomenological design is described as flexible and adapted to suit the phenomena under investigation. In this study, it is the cognitive effort of learners with cochlear implants (Holroyd 2001). The phenomenological design focuses on the experience in relation to what is under investigation. Interpretative phenomenological analysis was used to deeply explore and analyse the participants’ viewpoints and perceptions (Mole et al. 2019). Phenomenology was utilised as a theoretical framework found appropriate to the study as it provides the researchers with the opportunities to explore the unique psychological considerations of the research participants’ perceptions of cognitive effort (Murray & Holmes 2014).

### Participants

The participants were contacted through the Johannesburg Cochlear Implant Centre (JCIC). Purposive sampling was utilised to sample participants required for the study (Etikan, Musa & Alkassim 2016). To sample, the researcher selected certain participants who had the specific criteria for the study (Acharya et al. 2013; Etikan et al. 2016; Jawale 2012). The criteria included participants who were cochlear implant recipients, over 18 years of age and who must have already graduated from mainstream high schools. Participants younger than 18 years were excluded from the study. The participants included six cochlear implant recipients who attended mainstream schools in South Africa. All participants had graduated from their schools over the last eight years.

Participants were former learners with cochlear implants at mainstream schools. There were six participants. Three of them were female and between the ages of 21 and 25. The other three participants were male and between the ages of 24 and 27. The JCIC provided the researchers with a list of the participants who fit the criteria. The researchers then contacted the participants and each of them replied confirming their willingness to participate in the study. The researchers then asked them for personal details and requested that they read and sign the consent form. After consent was granted, semistructured interviews were conducted at convenient times.

### Data collection

Qualitative data collection methods supported the researchers to concentrate on the connotations of the data and to analyse through a critical and analytical approach (Noble & Smith 2014). To gather information on the experiences of the participants, semistructured interviews, which are qualitative data collection methods, were used (Guest, Namey & Mitchell 2013; Khan 2014; Noble & Smith 2014; Tolley et al. 2016). A total of six individual interviews were conducted from June 2020 to July 2020. Participants signed a consent form before the commencement of the interviews. All interviews were conducted through Zoom, an online communication platform, which was necessary as a result of the coronavirus disease 2019 (COVID-19) pandemic. The interviews lasted for 40 min – 55 min, depending on the participants’ responses. During the interviews, both the interviewer and interviewee enabled their camera functions so that they were able to view each other in order to make lip-reading possible, and the interviews were conducted in quiet spaces to avoid background noises. The interview guide was developed by the researchers beforehand, and it was used to gain information on the participants’ perspectives, as learners with cochlear implants, on the role of cognitive effort in their mainstream education. With consent acquired from the participants, the researchers utilised an audio-recorder to record the interviews.

### Data analysis

The researchers analysed the data according to the six phases of thematic analysis (Braun, Clarke & Weate 2016; Crowe, Inder & Porter 2015). Firstly, the researchers engaged deeply with the data and immersed themselves by reading it repeatedly. They read it several times to isolate the foundational connotations and trends. Then the researchers produced codes to identify the trends and themes and were proactive in searching for foundational and noteworthy ideas (Braun et al. 2016). Secondly, the researchers generated codes for the data. The researchers identified similarities and trends within the data (Braun et al. 2016). Thirdly, the researchers constructed themes from the data and categorised the data according to their respective themes (Braun et al. 2016; Crowe et al. 2015). Fourthly, the researchers reviewed the potential themes (Terry et al. 2017) and refined them. Fifthly, the researchers defined and named the themes. The significance and focus of the themes were clearly identified and discussed (Braun et al. 2016). Sixthly, the researchers produced a report based on the data (Terry et al. 2017). The discussions in the themes are linked to the interview transcripts (Braun et al. 2016) and specific events are chosen to display themes and connect the study to the data in the literature review (Braun et al. 2016). Five themes emerged from the analysis, namely auditory challenges, cognitive functioning, peer interactions, emotional health and concealed disability. In this study, we only present the theme of cognitive functioning, highlighting the experiences of cognitive effort and how it impacts learners with cochlear implants’ academic potential.

## Findings

Five themes emerged from the analysis. The themes were auditory challenges, cognitive functioning, peer interactions, emotional health and concealed disability. In the next section, we present data on cognitive effort of learners with cochlear implants in mainstream schools, as subthemes presented under the theme of cognitive functioning. These are experiences of cognitive effort, attention and cognitive effort and cognitive effort fatigue. Participants’ responses that related to the research objective were presented verbatim.

### Experiences of cognitive effort

When one is required to consciously engage in mental work, it is known as cognitive effort. Participants vocalised that they faced challenges with cognitive effort at their mainstream schools. They found themselves working harder than their hearing peers to decode spoken information. Some of the participants spoke about the extra effort it required for them to listen at school. The extra effort that they put into listening to the spoken information influenced their capabilities to multitask and retain information.

The understanding of this role was apparent in the following extracts from research participants:

‘I think because you have to, like, obviously listen a bit harder, whereas another person will just quickly pick up on the words that [*are*] being said and you have to actually concentrate harder to try and make out what the person is saying. And it can be, I do feel you have to concentrate a lot harder.’ (Jill, Graduated in 2013, Mainstream School)

‘I just think it comes naturally to deaf people or hard of hearing people that they will listen harder and concentrate a bit harder, even though it doesn’t show that they are doing it, but I think your internal is working harder.’ (Matthew, Graduated in 2013, Mainstream School)

Amy, a participant who graduated form her mainstream school in 2017 said that she frequently did additional schoolwork at home to make up for what she did not hear in the classroom. She reported, ‘I actually found myself in the end studying more than actually listening in class, a lot of times. Sometimes I have to self-study if I didn’t know what was going on’. She had to put in more effort to grasp spoken information, whereas her hearing peers needed to put in less effort in the same circumstance.

Matthew, a participant who graduated from a mainstream school in 2013 also commented, ‘because obviously I have to listen extra hard’. He expressed further that learners with cochlear implants are required to put in more cognitive effort to hear in class and it becomes natural for them to put in that additional cognitive effort. He said that it may not be noticeable to others, but internally, learners with cochlear implants work harder just to comprehend spoken language.

### Attention and cognitive effort

After rigorous analysis of the interview transcripts, two major themes emerged, namely attention and fatigue. The first major theme from this study was the role of attention in cognitive effort of learners with cochlear implants in mainstream schools. Attention is an integral part of learning at school. It provides the learners with the ability to focus on the task at hand. The participants found that they were encountering challenges in maintaining their attention in their mainstream classrooms.

One of the participants stated ‘I tend to get distracted quite easily sometimes’ (Peter, Graduated in 2013, Mainstream School). Another participant reported that his ‘attention just goes out of the window’ (Gary, Graduated in 2013, Mainstream School). One of the participants was provided with prescribed medication in high school to assist her challenges with concentration. She stated, ‘well, only later on in my life did I go on Concerta’. She described her attention span as ‘definitely in and out’. (Amy, Graduated in 2017, Mainstream School).

Another participant, Iris, said that she would easily lose focus in class when her attention was diverted by other noises and sounds:

‘I would definitely get distracted quite easily in class. Like I said earlier, I would hear all these different noises and then I would, you know, try and listen to that instead of listening to my teacher.’ (Iris, Graduated in 2013, Mainstream School).

One of the participants Jill, who graduated from a mainstream school in 2013 said: ‘my concentration span was pretty short. Even in a 30 min lesson, I wouldn’t be able to listen to the entire lesson’.

### Cognitive effort fatigue

The second major theme to emerge from this study was fatigue. Energy levels are an important factor for success at school. This is because good energy levels can assist learners in completing their required tasks and putting effort into their education. Some participants reported that they experienced additional exhaustion at their mainstream schools. As learners with cochlear implants, they found that they were more tired than their hearing peers.

A participant stated:

‘I definitely found that I was a lot more tired than my friends, and especially because, like, you have to concentrate to listen, whereas for them it’s like a natural thing.’ (Amy, Graduated in 2017, Mainstream School).

She said, ‘I had no energy or willpower to want to do homework, ever. Yeah, so that’s why I actually got the tutor, because I was feeling that [*I can’t do anything*]’. She explained, ‘I just felt I was getting a lot more tired because I wasn’t just concentrating on the work; I was concentrating to hear’.

Another participant named Jill and also graduated a main stream school in 2013 said, ‘I think my energy levels are generally quite low in school, compared with what they are now, for example’. She said that she ‘was tired a lot’. She expressed that having to apply additional effort in order to grasp everything said at school was a tiring experience. She explained it by saying, ‘it can be like exhausting, you know, having to really utilise your cochlear implants then’.

One of the participants, Matthew who graduated from a mainstream school in 2013 communicated. ‘I might be tired at the end of the day from listening’.

This section summarised and discussed the results of the thematic analysis. Two themes emerged. The themes were demonstrated by verbatim quotes of the participants. The results revealed that attention and fatigue contributed to the challenges that learners with cochlear implants encounter with cognitive effort at their mainstream schools. The participants discussed their challenges with cognitive effort and how they were required to put in more effort than their peers to understand verbal data. Participants often found themselves struggling to focus and also experienced fatigue in class.

## Discussion

Cognitive effort refers to conscious intellectual effort required to complete certain tasks. Effort refers to the amount one has to engage with tasks that are demanding in nature (Westbrook & Braver 2015). Cognitive effort is required from learners at school. Many situations in the classroom need high-level cognitive effort on the part of the learners (Jorgensen & Messersmith 2015). They need to put in a certain amount of cognitive effort to engage with the learning material and tasks.

This study found that learners with cochlear implants encounter challenges with cognitive effort in their mainstream schools. These learners, despite having sophisticated hearing technology, still face challenges with their hearing. Learners with cochlear implants obtain auditory stimulation from their cochlear implant devices, but not completely at the level that is considered normal (Nakeva von Mentzer 2014). Some challenges include noisy classrooms, as they find it difficult to isolate individual sounds (Dammeyer 2010; Hoffman et al. 2016). Another challenge is difficulty following when their teachers have accents different from their own.

In order to manage these challenging listening demands, learners with cochlear implants may be required to rely more on controlled cognitive effort towards the goal of understanding spoken information (Pichora-Fuller et al. 2016). The reason for this could be that they have to use additional cognitive effort to decode spoken information because the auditory information is not processed naturally for them.

Cognitive effort is a limited-capacity resource that is used with the intention of overcoming difficult listening demands (Pichora-Fuller et al. 2016). When one task becomes more demanding or challenging, in this case trying to comprehend speech, more cognitive effort is required to maintain scholastic achievements (Faulkner & Pisoni 2013). An increase in cognitive effort related to performing the primary task causes lower performance on the secondary task (Gosselin & Gagné 2010). This can compromise and challenge their cognitive effort capacities in the classroom, which in turn could impact their academic potential. From the interviews with the participants, two major themes emerged, namely attention and fatigue.

Attention was striking, as most of the participants reported to have faced challenges with their concentration span at their mainstream schools. These participants claimed that they struggled with maintaining their focus in the classroom and they were easily distracted. Regarding attention and concentration, findings from studies by Quittner et al. (2014) and Spencer and Marschark (2003) confirmed that learners with cochlear implants perform lower than average.

The participants communicated that they were often unable to maintain their concentration in class and therefore they would miss some of the lesson content. This could be because of the extra effort required by learners with cochlear implants to grasp spoken information at their mainstream schools. The reason for this could be that because their cognitive effort was already being overused and overworked, their concentration span was compromised. Mehrkian et al. (2019) stated that this causes attention and focus challenges. This affects learners with cochlear implants at school because they do not grasp information in class when they are not focused. This increases the pressure on them to catch up on what they missed. The requirement for additional cognitive effort also frequently causes exhaustion and lower energy levels.

Fatigue was another theme that emerged from the data. Watson, Verschuur and Lathlean (2016) stated that learners with cochlear implants often tend to feel tired and experience lower energy levels. Learners with cochlear implants, because of the additional cognitive effort they need to use in order to process spoken information, get tired easier and more quickly. Learners with cochlear implants seem to be at greater risk for experiencing fatigue and low energy levels (Hornsby & Kipp 2016). This may be caused by their challenges in processing auditory signals, including spoken language (Hornsby & Kipp 2016).

Purdy et al. (2017) and Mehrkian et al. (2019) stated that learners with cochlear implants need additional cognitive effort to decode auditory data, and more energy is utilised to decipher it. Some of the participants reported that it was very tiring for them to put in the extra effort to listen all day at school. They experienced more exhaustion than their fellow hearing peers. The additional cognitive effort that was needed by these learners to hear information in class and to decode spoken information fatigued them during and after school. This impacts learners with cochlear implants at school because it is more difficult for them to work and complete tasks effectively with lower energy levels. This may lead these learners to not reaching their academic potential to the fullest (Mehrkian et al. 2019).

The effects of cognitive effort on academic performance could manifest in increased self-motivation of the learners with cochlear implants (Kuldas et al. 2014). Increased cognitive effort is expected to positively impact academic performance (Bircan & Sungur 2016). Although it may be strenuous for students with cochlear implants to increase their cognitive effort in order to achieve their learning goals, it appears imperative that they do. Academic performance that is positively influenced by cognitive effort depends mainly on the motivation to succeed. Techniques such as self-study add to the effort to succeed.

To conclude, learners with cochlear implants are required to use more cognitive effort to listen to and to grasp spoken information in their mainstream classrooms. This causes them to face challenges with their attention and focus (Mehrkian et al. 2019). In addition, these learners also end up feeling more fatigued than their peers (Watson et al. 2016).

## Implications

This study provides insights that would be an asset and advantage for teachers of learners with cochlear implants and other stakeholders. The studies that have been conducted in South Africa so far have mainly concentrated on hearing-impaired learners’ experiences at school. Research that has focused directly on learners with cochlear implants in mainstream schools in South Africa is very limited. Furthermore, the research on the cognitive effort, attention span and fatigue experienced by learners with cochlear implants is also generally limited.

### Recommendations for practice

In order to address the cognitive effort challenges of learners with cochlear implants, the following strategies are recommended:

- It would be beneficial for the learner with cochlear implants to be provided with the teacher’s notes in written form, even if summarised beforehand. This could be done instead of these students having to listen and write down the dictated information. Whilst the hearing peers take down the dictated information, the learners with cochlear implants can follow and highlight the printed notes. This helps the learners because not much cognitive effort will be expended rapidly.
- If the option of printed notes is not available or possible, the teacher could scan and check the lesson notes taken by the learner with cochlear implants to confirm their completeness and correctness. This would help the learners in case their cognitive effort, energy levels or attention dwindled during the class.
- Teachers should be conscious that the learner with cochlear implants may experience fatigue. It would be useful to reduce the time the learners attend school or participate in class. This would assist their energy levels.
- During the lesson, the teacher could make subtle checks on the learner and maintain awareness and understanding of the learners’ concentration, energy levels and management of the lesson’s content.
- It is recommended that a buddy system be established for the learners with cochlear implants. Another learner could be selected to help in each subject. The buddy should be seated next to the learner to give support during class. It is recommended that various buddies be selected and spread over different subjects to reduce the responsibility being overwhelming. This would decrease the pressure on the teachers and provide the learner with cochlear implants with the additional support.

### Recommendations for policy

Learners with cochlear implants have been educated in mainstream schools despite their hearing impairment. Despite this, teachers in mainstream schools have usually not been trained specifically to help and assist these learners:

- It is recommended that policies incorporate the training needed for teachers and educators of learners with cochlear implants. School policies should encompass and implement the necessary support for these learners.
- Mainstream schools should be familiar with the cognitive effort challenges that learners with cochlear implants face in the classroom. They should also understand the challenges these learners face regarding attention and fatigue.
- All educators should have sufficient knowledge of policies that support these learners so that they are able to reach their full academic potential.

### Recommendations for research

This research explored how cochlear implant recipients experienced cognitive effort challenges whilst attending their mainstream schools. Each participant in this study graduated from their mainstream schools within the last eight years. The technical advances in the cochlear implant technology have not been as many since then. Therefore, research could direct its focus on participants who have completed their mainstream schooling more recently. Researchers could also aim to direct their research to recipients of cochlear implants who are students at mainstream schools currently.

The participants of this study were recipients of cochlear implants who were learners who graduated from mainstream schools. Future research could focus on other participants. Some examples would be health professionals who work directly with these learners, such as audiologists and speech therapists. Ear, nose and throat (ENT) doctors who perform cochlear implant surgeries could also be incorporated in research. Researchers could also involve the parents and families of these learners in the research to obtain more information.

Research could focus on the coping mechanisms of learners with cochlear implants. Frequently, learners with cochlear implants are not aware of how to manage in their mainstream schools regarding cognitive effort, attention and fatigue. They are also frequently unaware of the accommodations from which they can benefit. Various health professionals (such as audiologists, speech therapists and educational psychologists), trained educators and previous graduates who are recipients of cochlear implants could give these learners the required assistance and tools.

## Limitations

This study had a small sample size of six participants. Furthermore, only one data collection method, the semistructured interviews, was utilised. Therefore, this study cannot be generalised to other contexts and circumstances. However, qualitative studies are not supposed to have large samples (Holloway & Galvin 2016). In addition, they provide in-depth information (Given 2015) by utilising the perceptions and observations of the participants through the interpretive paradigm and method.

## Conclusion

Learners with cochlear implants encounter challenges with cognitive effort in mainstream schools. Cognitive effort impacts these learners in two different ways. The first is their attention. Learners with cochlear implants lose focus and get distracted easily. This is because their cognitive effort is compromised by having to constantly decode spoken information, which they do not do naturally. The second is fatigue. As a result of the additional cognitive effort that learners with cochlear implants need to put into listening in class, learners become exhausted. This study showed that in order to assist learners with cochlear implants at mainstream schools, they require interventions that help them manage the cognitive effort they are using in their classrooms.

The findings of this study could contribute to general awareness of the challenges that learners with cochlear implants encounter in mainstream schools. It is recommended that teachers of learners with cochlear implants receive training on assisting these learners in managing challenges with cognitive effort. This study could also be used as a guide for the learners themselves to manage the cognitive effort challenges that they encounter. They would also find value and assistance from the recommendations provided in the study. This study is a crucial advancement towards the inclusion of learners with cochlear implants in South Africa. It is desired that it will be a small contribution towards the acknowledgement and understanding of the academic potential of learners with cochlear implants and how to support them in mainstream schools.
